# miR-Blood – a small RNA atlas of human blood components

**DOI:** 10.1038/s41597-024-02976-z

**Published:** 2024-02-02

**Authors:** Julia Jehn, Franziska Trudzinski, Rastislav Horos, Judith Schenz, Florian Uhle, Markus A. Weigand, Maurice Frank, Mustafa Kahraman, Marco Heuvelman, Tobias Sikosek, Timothy Rajakumar, Jennifer Gerwing, Jasmin Skottke, Alberto Daniel-Moreno, Christina Rudolf, Franziska Hinkfoth, Kaja Tikk, Petros Christopoulos, Laura V. Klotz, Hauke Winter, Michael Kreuter, Bruno R. Steinkraus

**Affiliations:** 1 Hummingbird Diagnostics GmbH, Im Neuenheimer Feld 583, 69120 Heidelberg, Germany; 2 Center for Interstitial and Rare Lung Diseases, Thoraxklinik, University of Heidelberg, and German Center for Lung Research (DZL), Heidelberg, Germany; 3 Department of Anesthesiology, Medical Faculty, Heidelberg University, Heidelberg, Germany; 4 Department of Thoracic Oncology, Thoraxklinik, University of Heidelberg, Translational Lung Research Center Heidelberg (TLRC-H), and German Center for Lung Research (DZL), Heidelberg, Germany; 5 Department of Thoracic Surgery, Thoraxklinik, University of Heidelberg, Translational Lung Research Center Heidelberg (TLRC-H), and German Center for Lung Research (DZL), Heidelberg, Germany

**Keywords:** Small RNAs, miRNA in immune cells

## Abstract

*miR-Blood* is a high-quality, small RNA expression atlas for the major components of human peripheral blood (plasma, erythrocytes, thrombocytes, monocytes, neutrophils, eosinophils, basophils, natural killer cells, CD4+ T cells, CD8+ T cells, and B cells). Based on the purified blood components from 52 individuals, the dataset provides a comprehensive repository for the expression of 4971 small RNAs from eight non-coding RNA classes.

## Background & Summary

Small non-coding RNAs (sRNAs) are often tissue or even cell type specific and their expression profiles can change under pathologic conditions. sRNAs that are secreted or released into the extracellular space are stable in blood and other body fluids. This has opened exciting opportunities for their diagnostic use through minimally invasive ‘liquid biopsies’ which can be analyzed by PCR or NGS. Several sRNA biomarkers are in clinical development offering the potential to improve patient management from early cancer detection to immuno-oncology response prediction^1–7^.

To chaperone the translation of sRNA-based diagnostics from bench to bedside, cell-specific expression data is required to elucidate biomarker origin and generate mechanism of action hypotheses. sRNA analyses are typically performed on (i) plasma/serum, (ii) extracellular vesicles or (iii) unfractionated whole blood collected via stabilization tubes, and only rarely on purified cell populations. In the case of whole blood collection, blood cells are immediately lysed upon contact with the stabilization reagent and the *post hoc* attribution of sRNA signal to its respective sources is no longer possible. However, pinpointing the cellular compartment which underlies the differential expression of the sRNA of interest will offer additional insights into the biology of the biomarker (e.g. implicated in innate or adaptive immunity?) and is important to guide downstream functional studies.

**Fig. 1:**
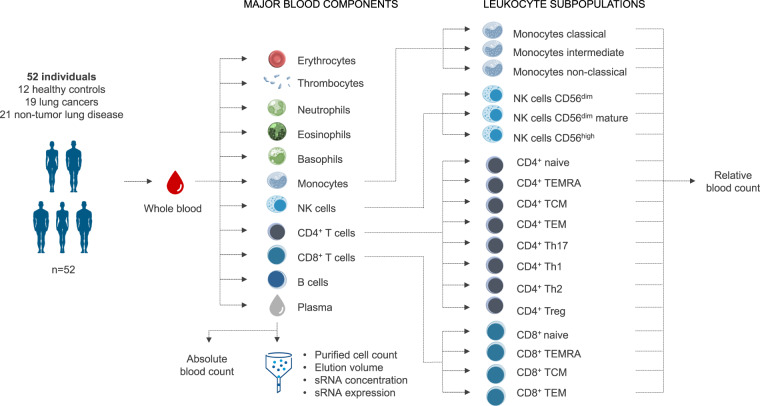
Overview of the study cohort and samples. From 52 human individuals of a mixed cohort (healthy and lung diseases), the eleven major components of blood were purified. For each purified blood component and a whole blood sample of the same donor, the sRNA population was isolated and subjected to sRNA sequencing for expression analysis. For the individual purified blood fractions of each donor, we additionally obtained the cell counts, elution volume and sRNA concentration to determine the sRNA content of each sample. Furthermore, the relative blood counts of 18 subpopulations of monocytes, NK cells, CD4+ T cells, and CD8+ T cells were determined. Images of the blood cell types were modified from Häggström^22^.

To this end, several expression datasets have been generated that offer sRNA profiles of tissue and cell types^8,9^. However, the currently available studies on human peripheral blood are typically siloed (e.g. one cell type only), and dedicated resources are either microRNA (miRNA) focused^10^, omit important blood cell types (e.g. myeloid cells), or are not interactively explorable^11^. We here present a comprehensive sRNA expression resource with matched blood count and cell sorting metrics (Fig. 1) for the eleven dominant blood components (plasma, erythrocytes, thrombocytes, monocytes, neutrophils, eosinophils, basophils, natural killer cells, CD4+ T cells, CD8+ T cells, and B cells). The processed dataset contains expression data for 4971 sRNAs from eight non-coding RNA families: miRNAs, transfer RNAs (tRNAs), ribosomal RNAs (rRNAs), long non-coding RNAs (lncRNAs), small nucleolar RNAs (snoRNAs), small nuclear RNAs (snRNAs), Y RNAs, and PIWI-interacting RNAs (piRNAs).

**Fig. 2:**
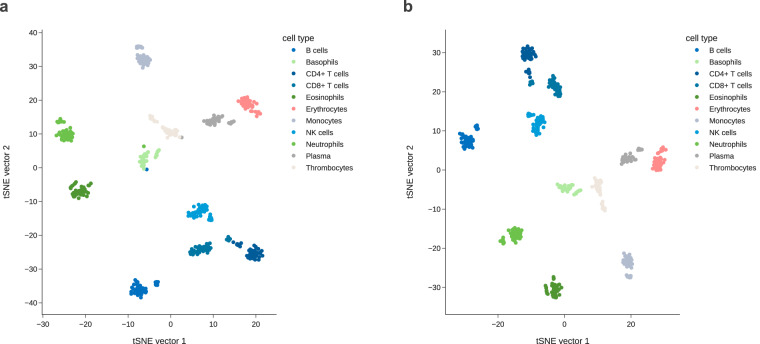
t-SNE plot of normalized expression data. The samples were coloured by its blood component group. (**a**) t-SNE plot of the 514 blood component specific samples with adequate purity (purity > 70% as determined by flow cytometry) shows that four samples do not cluster with the samples of the same blood component. For further analyses these samples were removed. (**b**) The t-SNE plot of the 510 blood component specific samples passing the quality control filters shows clearly defined clusters per blood component.

Generated based on donated blood from 52 individuals in a mixed cohort (healthy and lung diseased individuals) with a stringent quality filter regime applied at all stages of sample processing and data preparation, the dataset is a robust resource with low sample-to-sample variability per blood component (Fig. 2b). In comparison to the previous benchmark^10^, our human sRNA blood component atlas includes sRNAs mapping to longer RNA transcripts or precursors of seven RNA classes in addition to miRNAs and covers three additional blood components (thrombocytes, basophils, and eosinophils). It therefore represents the most comprehensive collection of human sRNA expression data for isolated blood components to date. To facilitate querying the dataset, we developed *miR-Blood*, an interactive and user-friendly dashboard. The dashboard is available at http://mir-blood.com/.

## Methods

### Ethics declaration

The two studies on which the data are based were approved by the Heidelberg University ethics committee of the medical faculty (S-916/2019 and S-551/2020) and registered in the German Clinical Trials Register (DRKS) under DRKS00022300 on 2020/06/29 and DRKS00023138 on 2020/09/25. All patients provided written informed consent, including for data sharing. We hereby confirm that we have complied with all relevant ethical regulations.

### Blood donor cohort and study samples

Approximately 100 ml whole blood was drawn into EDTA tubes from 52 donors. 12 of them were healthy donors, 19 were diagnosed with lung cancer and 21 with a non-malignant lung disease. An overview on the age and gender distribution of the study participants is listed in Table 1. The blood was directly used for cell sorting and plasma purification, resulting in eleven fractional derivatives per sample (Fig. 1, middle part). For each blood donor the sRNA population was additionally analysed directly from unfractionated whole blood collected in S Monovette EDTA K3 tubes (Sarstedt AG & Co. KG, Nümbrecht, Germany).

**Table 1:** Donor overview showing the age and gender distribution for each disease group.

	N	Gender, n (%)		Age, mean (SD)
		Female	Male	
**Overall**	52	30 (58%)	22 (42%)	63 (8)
**Healthy**	12	6 (50%)	6 (50%)	55 (3)
**Lung Cancer**	19	11 (58%)	8 (42%)	67 (8)
**Non-malignant Lung Disease**	21	13 (62%)	8 (38%)	63 (7)

### Blood count data

**Fig. 3:**
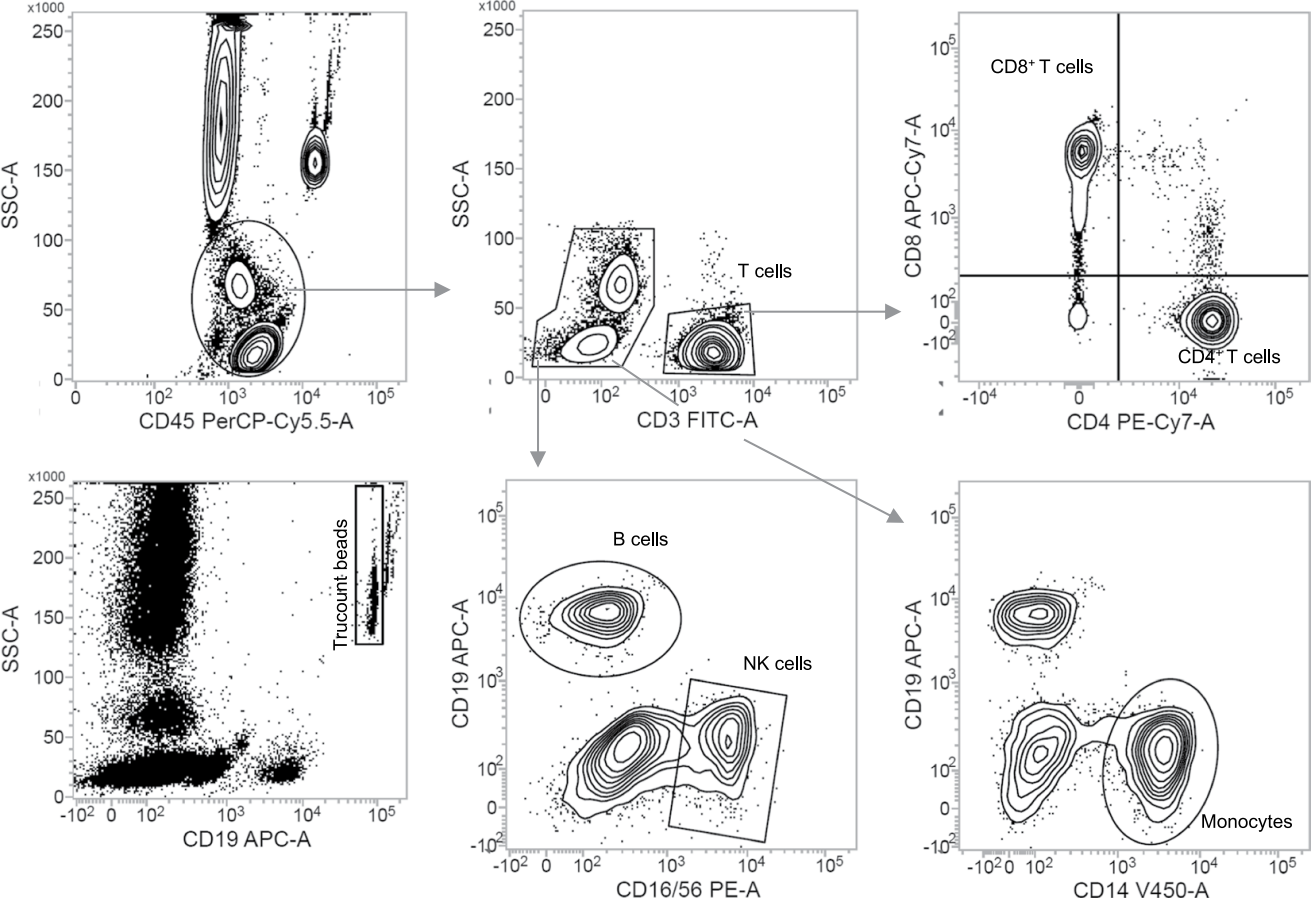
Representative gating strategy absolute counts of lymphocyte subsets and monocytes. Quantification of lymphocyte subsets and monocytes using BD Multitest 6-color TBNK reagent and anti-Human CD14-V450. Trucount beads were used to determine absolute counts.

**Fig. 4:**
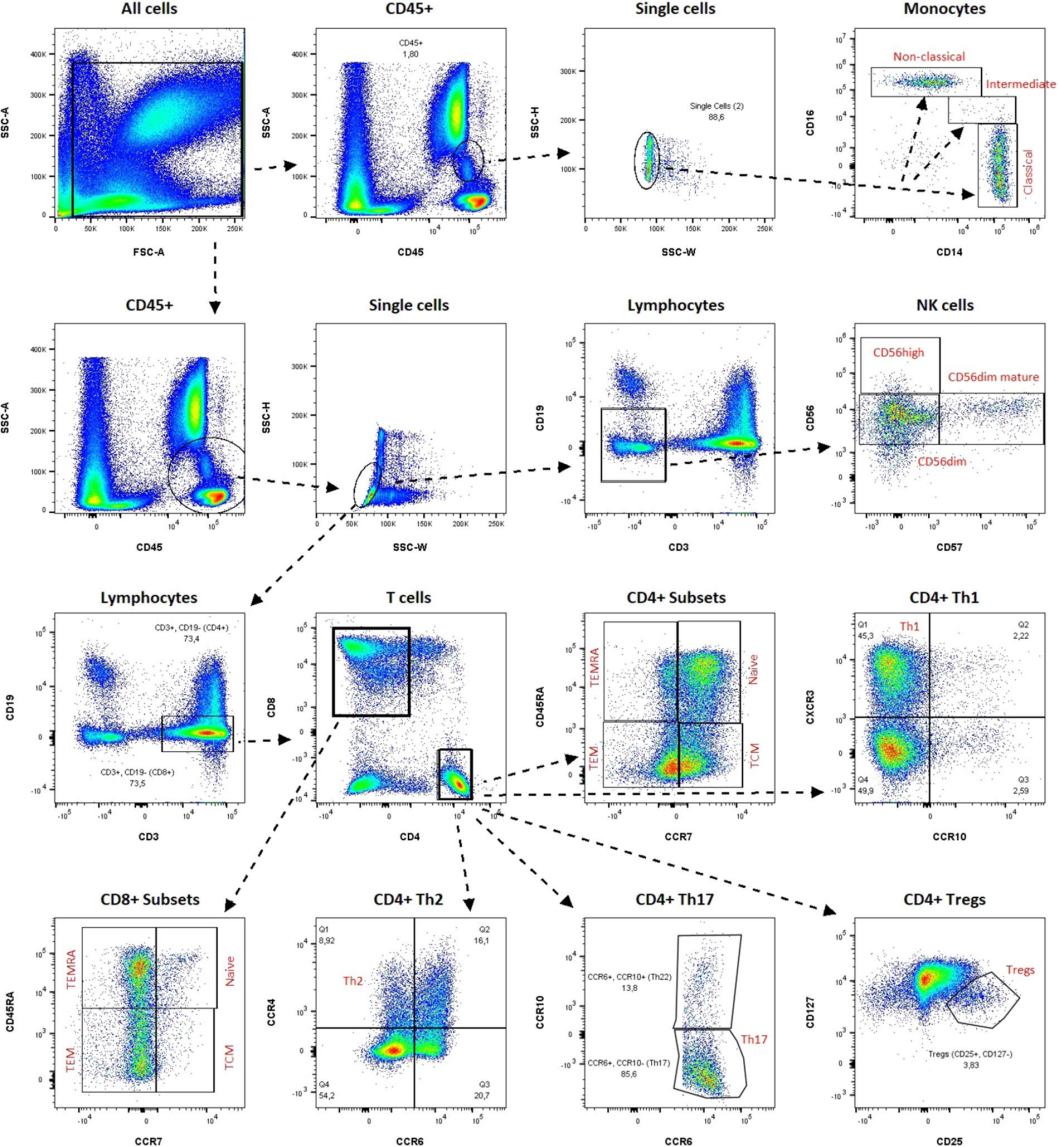
Representative gating strategy relative counts of 18 leukocyte subpopulations. Relative cell counts were turned into absolute values by multiplying the absolute cell counts of the respective parent cell population.

Absolute erythrocyte, thrombocyte, neutrophil, eosinophil, and basophil counts were obtained from clinical differential blood counts. Absolute cell counts of CD4^+^ T cells, CD8^+^ T cells, B cells, NK cells, and monocytes, were determined by flow cytometry analysis. For this purpose, 50 µl of the whole blood samples were stained with the BD Multitest 6-color TBNK reagent in combination with anti-Human CD14-V450 (clone: MϕP9) in BD Trucount tubes and measured on a either FACSVerse or a FACSLyric flow cytometer. Absolute cell counts were quantified on basis of the Trucount beads using the BD FACSuite software (all BD Biosciences, Franklin Lakes, NJ, USA). A representative gating strategy is shown in Fig. 3. To further quantify multiple leukocyte subpopulations, 100 µl of the same whole blood samples were stained with 17 antibodies (CD16, CD3, CD197 (CCR7), CD57, CD25, CD194 (CCR4), CD127, CD8, CD196 (CCR6), CD56, CD45RA, CD4, CD45, CD19, CCR10, CD14, CD183 (CXCR3)) from the BD 27-colour broad phenotyping panel (BD Biosciences, Franklin Lakes, NJ, USA) and measured on a FACSymphony flow cytometer. A representative gating strategy is shown in Fig. 4. Relative quantification was performed using the BD FlowJo software. The relative frequencies obtained for these sub cell type populations were turned into absolute cell counts per blood volume by multiplying the absolute cell counts of the respective parent cell population. The blood counts of the ten major blood cell types and the 18 leukocyte subpopulations are listed in Supplementary Table 1.

### Cell sorting of blood cells

Cell sorting was performed immediately after drawing of human whole blood. CD4+ T cells, monocytes, and B cells were isolated and separated from whole blood. Therefore, 350 µl from the MicroBeads of the corresponding whole blood isolation kit were added to 7 ml whole blood and incubated 15 min at 4 °C. Afterwards the cells were washed with 43 ml isolation buffer (0.5% w/v albumin [Carl Roth GmbH & Co KG; Karlsruhe Germany], 2 mM ethylenediaminetetraacetic acid in PBS) and centrifuged 10 min at 445 g and room temperature (RT). The pellet was resuspended in 1 ml isolation buffer and separated using positive selection on autoMACS Pro Separator (Miltenyi Biotec GmbH, Bergisch Gladbach, Germany). For the isolation of CD8+ T cells, neutrophils, and natural killer (NK) cells, the corresponding MACSxpress whole blood isolation reagent (see Table 2) was added in a ratio of 1:2 to the whole blood. Next, the tubes were positioned in a MACSmix Tube Rotator (Miltenyi Biotec GmbH) for 5 minutes at room temperature. Unwanted cell populations were labelled with the respective kits (see Table 2). After 15 minutes at RT on a MACSxpress Separator (Miltenyi Biotec GmbH), the CD8+ T cells were purified through a second negative selection on the MACSxpress Separator. To purify neutrophils and NK cells, lysis of erythrocytes was performed by adding 20 ml 0.2% sodium chloride solution (Merck KGaA, Darmstadt, Germany) for 20 seconds and 20 ml 1.6% sodium chloride solution sequentially. Next, the cell suspensions were centrifugated for 5 minutes at 300 g and 4 °C, and the supernatant was discarded.

**Table 2:** List of kits used to purify the major blood cell types.

Cell type	Kit name (Miltenyi Biotec, Bergisch Gladbach, Germany)
**Monocytes**	StraightFrom® Whole Blood CD14 MicroBeads
**B cells**	StraightFrom® Whole Blood CD19 MicroBeads
**CD8+ T cells**	StraightFrom® Whole Blood CD8 MicroBeads or MACSxpress® Whole Blood CD8 T cell Isolation Kit
**CD4+ T cells**	StraightFrom® Whole Blood CD4 MicroBeads
**Erythrocytes**	CD235a (Glycophorin A) MicroBeads
**Thrombocytes**	CD61 MicroBeads
**Basophils**	Diamond Basophil Isolation Kit
**Eosinophils**	Eosinophil Isolation Kit
**Neutrophils**	MACSxpress® Whole Blood Neutrophil Isolation Kit
**NK cells**	MACSxpress® Whole Blood NK Cell Isolation Kit

To isolate human thrombocytes, basophils, and eosinophils, the whole blood was diluted in a ratio of 2:3 with phosphate-buffered saline (PBS) (Thermo Fisher Scientific) and layered over the density gradient medium Histopaque 1077 (Merck KGaA) in a ratio of 3:5. After a centrifugation for 20 minutes at 600 g and RT, the different layers were isolated immediately.

The uppermost layer was used to isolate thrombocytes. After an additional centrifugation step for 15 minutes at 500 g and RT, the supernatant was discarded, and the pellet was resuspended in 600 μl isolation buffer, and 150 μl CD61 beads were added (see Table 2). After an incubation time of 15 minutes at 4 °C, the pellet was washed and then resuspended in 500 μl isolation buffer, and the thrombocytes were isolated with the autoMACS Pro Separator.

The ring-like sediment at the interface between the uppermost and the Histopaque 1077 layer was washed three times with the isolation buffer. The isolation of basophils was performed in a two-step procedure with the Diamond Basophil Isolation Kit (see Table 2). First, the cell pellet was resuspended in 300 μl isolation buffer, 100 μl FcR Blocking Reagent, and 100 μl Basophil Biotin-Antibody Cocktail. After incubating for 10 minutes at 4 °C, 300 μl isolation buffer and 200 μl Anti Biotin MicroBeads were added. After incubating again for 10 minutes at 4 °C, cell suspension was washed. Then, the washed cell pellet was resuspended in 500 μl isolation buffer, and basophils were isolated with the autoMACS Pro Separator (negative selection). Afterward, the enriched basophils were washed. The resulting cell pellet was resuspended in 100 μl CD123 MicroBeads and incubated for 15 minutes at 4 °C. Then, the cell suspension was washed, and the resulting cell pellet was resuspended in 500 μl isolation buffer. Last, magnetic labelled basophils were isolated with the autoMACS Pro Separator (positive selection).

The bottom layer was purified by performing several erythrocyte lysis steps. Next, the cell suspension was centrifugated for 5 minutes at 300 g and 4 °C. The pellet was then resuspended in 40 μl isolation buffer per 10^7^ cells and in 10 μl eosinophil biotin-antibody cocktail per 10^7^ cells (see Table 2). After an incubation time of 10 minutes at 4 °C, 30 μl isolation buffer per 10^7^ cells and 20 μl Anti Biotin MicroBeads per 10^7^ cells were added to the cell suspension, incubated 15 minutes at 4 °C, washed, and finally resuspended in 500 μl isolation buffer. Eosinophils were isolated with the autoMACS Pro Separator (negative selection).

To separate the erythrocytes from human whole blood, 5 ml whole blood was centrifuged for 10 minutes at 2500 g and RT. The plasma was centrifuged again to remove residual erythrocytes for 2 minutes at 13,000 g and RT, and 1 ml aliquots were frozen at −80 °C. The remaining pellet of the first centrifugation was resuspended in isolation buffer. The cell suspension was filtered with a 40 μm cell strainer (Greiner Bio-One GmbH, Frickenhausen, Germany). The filtered cell suspension was diluted in a ratio of 1:3 with the isolation buffer. After cell counting, approximately 3 × 10^7^ erythrocytes were used for further processing. Next, this cell suspension was centrifuged for 10 minutes at 300 g and 4 °C. Afterward, the cell pellet was resuspended in 240 μl isolation buffer and in 60 μl CD235a MicroBeads (see Table 2) and incubated for 15 minutes at 4 °C. After washing, the cell pellet was resuspended in 150 μl isolation buffer. Last, magnetically labelled erythrocytes were isolated with the autoMACS Pro Separator (positive selection).

In cases where the provided blood volume was too little, the purification of lymphocytes was prioritized over thrombocytes, eosinophils, and basophils resulting in 539 fractional blood derivatives overall.

### Quality control of sorted cells

**Fig. 5:**
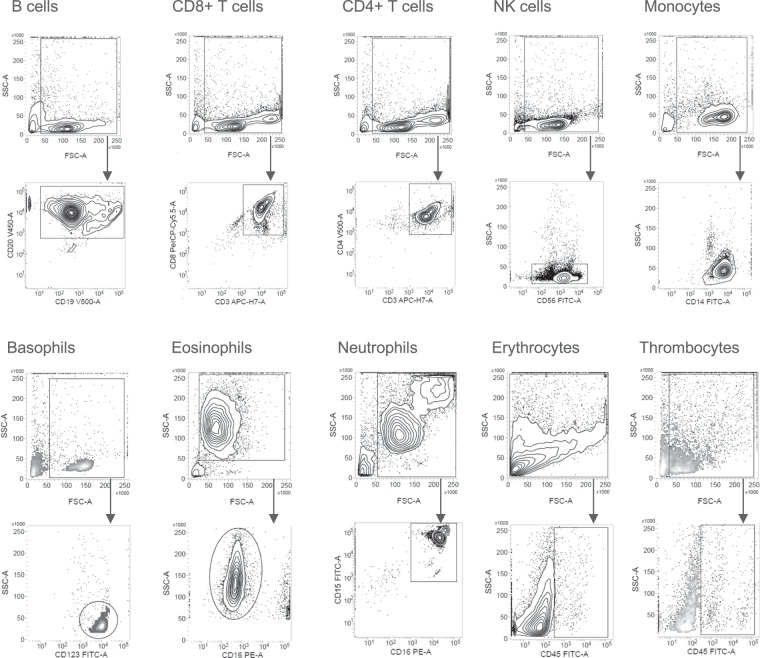
Representative gating strategy flow cytometry quality control measured on a FACSVerse machine. Cellular events were gated from all events. B cells were identified as CD20+. CD8+ T cells were identified as CD3+ CD8+. CD4+ T cells were identified as CD3+ CD4+. NK cells were identified as CD56+. Monocytes were identified as CD14+. Basophils were identified as CD123+. Eosinophils were identified as CD16-. Neutrophils were identified as CD15+ CD16+. Erythrocytes were identified as CD45-. Thrombocytes were identified as CD45−.

**Fig. 6:**
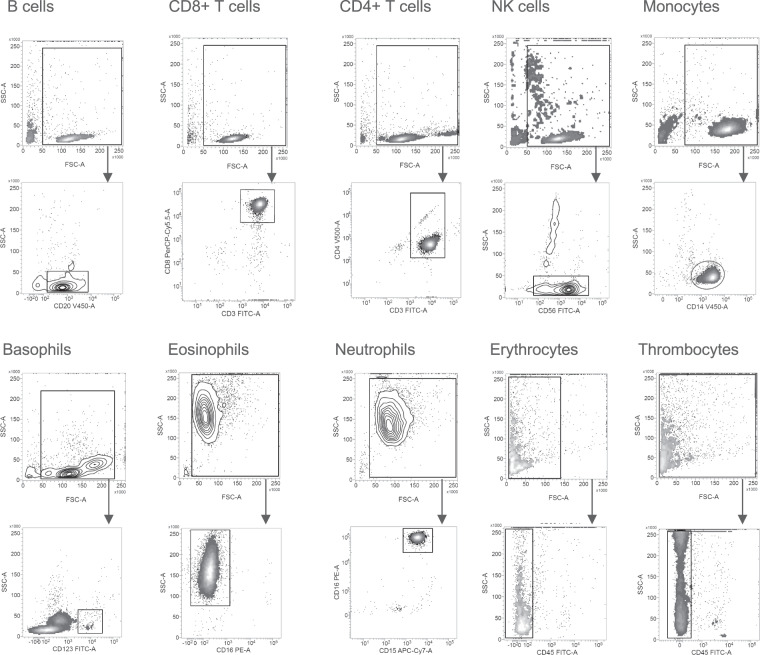
Representative gating strategy flow cytometry quality control measured on a FACSLyric machine Cellular events were gated from all events. B cells were identified as CD20+. CD8+ T cells were identified as CD3+ CD8+. CD4+ T cells were identified as CD3+ CD4+. NK cells were identified as CD56+. Monocytes were identified as CD14+. Basophils were identified as CD123+. Eosinophils were identified as CD16-. Neutrophils were identified as CD15+ CD16+. Erythrocytes were identified as CD45−. Thrombocytes were identified as CD45−.

For each purification, yield and purity of isolated cells was determined by flow cytometry using a small aliquot of the sample. Approximately 0.5 × 10^5^ cells of each population were diluted in 2 ml isolation buffer and centrifuged afterward for 5 minutes at 300 × g and RT. Next, the supernatants were decanted, and the cell pellets were resuspended in 100 μl isolation buffer. After adding 5 μl Human TruStain FcX (Fc Receptor Blocking Solution) (BioLegend; San Diego, CA), the cell homogenates were incubated for 10 minutes at RT. To stain the different cell populations, the corresponding antibodies were added (see Table 3). After incubating for 20 minutes at 4 °C in the dark, 2 ml isolation buffer was added, and the cell homogenates were centrifugated for 5 minutes at 300 g and RT. Then, the cell pellets were resuspended in 300 μl isolation buffer. A FACSLyric flow cytometer was used for all measurements. Results were analysed using BD FACSuite software (both from BD Biosciences, Franklin Lakes, NJ). Representative gating strategies are shown in Figs. 5, 6. Purity of erythrocytes and thrombocyte preparations was evaluated by unwanted presence of CD45+ cells. For 25 cellular fractions (4.6% of derivatives, 25/539) the purification process did not meet the prespecified quality standard (purity >70% as determined by flow cytometry). These samples were excluded from further analyses. The measured cell sorting metrics of the purified blood component samples are listed in Supplementary Table 2.

**Table 3:** List of antibodies used for quality control flow cytometry.

Cell type	Reagent	Reference number	Supplier
Basophils	FITC Mouse Anti-Human CD123	558663	Becton, Dickinson and Company; Franklin Lakes (New Jersey), USA
B cells	V500 Mouse Anti-Human CD19	561121	Becton, Dickinson and Company; Franklin Lakes (New Jersey), USA
	V450 Mouse Anti-Human CD20	561164	Becton, Dickinson and Company; Franklin Lakes (New Jersey), USA
CD8+ T cells	FITC Mouse Anti-Human CD3	555332	Becton, Dickinson and Company; Franklin Lakes (New Jersey), USA
	PerCP-Cy^TM^5.5 Mouse Anti-Human CD8	565310	Becton, Dickinson and Company; Franklin Lakes (New Jersey), USA
Eosinophils	FITC Mouse Anti-Human CD16	561308	Becton, Dickinson and Company; Franklin Lakes (New Jersey), USA
Erythrocytes	FITC Mouse Anti-Human CD45	555482	Becton, Dickinson and Company; Franklin Lakes (New Jersey), USA
Monocytes	V450 Mouse Anti-Human CD14	560349	Becton, Dickinson and Company; Franklin Lakes (New Jersey), USA
Neutrophils	APC/Cy7 Anti-Human CD15	323048	BioLegend; San Diego, USA
	FITC Mouse Anti-Human CD16	561308	Becton, Dickinson and Company; Franklin Lakes (New Jersey), USA
NK cells	FITC Mouse anti-Human CD56	562794	Becton, Dickinson and Company; Franklin Lakes (New Jersey), USA
CD4+ T cells	FITC Mouse Anti-Human CD3	555332	Becton, Dickinson and Company; Franklin Lakes (New Jersey), USA
	V500 Mouse Anti-Human CD4	560768	Becton, Dickinson and Company; Franklin Lakes (New Jersey), USA
Thrombocytes	FITC Mouse Anti-Human CD45	555482	Becton, Dickinson and Company; Franklin Lakes (New Jersey), USA

### RNA isolation and generation of sRNA libraries

The cell and plasma fractions were directly lysed in Qiazol, as were the whole blood samples. For RNA isolation the miRNeasy Serum/Plasma kit with minElute columns (Qiagen, Venlo, Netherlands) was used. From the obtained total RNA, the “sRNA concentration” of the 10 to 200 nucleotide region and the “microRNA concentration” of the 10 to 40 nucleotide region was determined on a Fragment Analyzer (Agilent, Santa Clara, USA) and is listed in Supplementary Table 2. (Dual) unique indexed sRNA libraries with unique molecular identifiers (UMIs) adaptors were generated using the QIAseq® miRNA Library Kit (Qiagen, Venlo, Netherlands). The library prep were performed according manufacturer’s instructions. For ligation, 100 ng of total RNA, or maximal 5 μl of RNA if concentration was not sufficient, was used. When this caused less input of only 50 ng or 10 ng, adapter and RT primer were prediluted 1:2.5 or 1:5, respectively. Mag-Bind beads (Omega Bio-Tek, Norcross, GA) were used for RT clean-up using 2.5x ratio to sample according to manufacturer’s protocol. Library polymerase chain reaction (PCR) was performed using custom dual index primers or QIAseq miRNA 96 Index IL (MIHT1-96). PCR was cleaned-up with Mag-bind beads using 0.9 × and 1.8 × ratios. DNA concentration was determined using QuantIT kit (ThermoFisher Scientific, Waltham, MA) on VictorNivo plate reader (PerkinElmer, Waltham, MA) or KAPA Library Quantification kit (Roche Holding AG, Basel, Switzerland) on QuantStudio 6Flex (ThermoFisher Scientific, Waltham, MA). After controlling the library quality on a Fragment Analyzer (Agilent, Santa Clara, USA), samples with high adapter-dimer concentration were loaded on a Novex 8% TBE gel (ThermoFisher Scientific, Waltham, MA) and the area above 180 base pair was excised. These libraries were eluted from gel overnight at 37 °C and gel particles were removed with Corning™ Costar™ spin-X centrifuge tubes (Corning, New York, US). Libraries were purified using DNA Clean and Concentrator kit (Zymo Research, Freiburg, Germany), or NEXTflex clean-up beads (PerkinElmer, Waltham, US). Finally, equimolar library pools with up to 192 samples were prepared.

### sRNA sequencing and data processing

For sequencing, multiplexed library pools were adjusted to 0.5 nM with resuspension buffer (10 mM Tris-HCl, pH 8.5 with 0.1% Tween 20) in 24 µl containing 0.5 nM PhiX and loaded on Illumina NextSeq. 2000 (Illumina, San Diego USA). For index2 a custom primer was used in a concentration of 0.6 µM in 500 µl HT1 buffer (Illumina, San Diego USA) and added to the custom well in the reagent cartridge. The NextSeq. 2000 P3 Reagent Cartridge (88 cycles) were used with 71 Read1 cycles and 8 cycles for index1 and 2. For sequencing with Illumina NextSeq. 500 library pools were adjusted to 15–60 nM. Raw sequence reads were adapter trimmed and depleted of PCR duplicates based on the UMIs using a customized R-based pre-processing pipeline. Only sequences that had at least one read in at least three samples per blood component type and that were longer than 17 nucleotides were included in the count matrix. To annotate the pre-processed sRNA sequences, the annotation pipelines unitas based on SeqMap and SPORTS based on Bowtie were used^12–15^. With each tool the respective pre-compiled reference databases were used together with the snoDB resource v2.0 as additional snoRNA reference. The used mapping reference per sRNA class is listed in Table 4. With unitas, miRNA mapping was restricted to human hairpin sequences allowing for two non-templated 3’ nucleotide additions and one internal modification. Mapping to other non-miRNA references was restricted to a maximum of one mismatch and did not allow for insertions or deletions. With SPORTS, only one mismatch in the entire alignment was allowed. For the sRNA classes rRNA and Y RNA we used the annotation labels provided by SPORTS. For the other six sRNA classes (miRNA, tRNA, snoRNA, lncRNA, snRNA, piRNA) the annotation labels provided by unitas were used. In case of multi-assignments, the annotation was prioritized in the following manner: miRNA > tRNA > rRNA > Y RNA > snoRNA > lncRNA > snRNA > piRNA. To subcategorize rRNA- and Y RNA-derived sRNAs further, their parental RNA sequences were binned to ~ 25 nucleotide bins. The annotation label is then issued as a combination of the gene symbol of the parental RNA to which the sequence maps and the number of the bin which contains the starting position of the sRNA sequence. Finally, the reads are collapsed based on the annotation labels of the eight non-coding RNA classes and normalized for library size as reads per million (RPM). For visualization purposes the RPM values were log2-transformed (log2(RPM + 1)). Low expressed sRNAs (RPM values < 2) were discarded from the dataset.

**Table 4:** Used reference databases for mapping of sRNA sequencing reads.

sRNA classes	Database (Release); Reference
**miRNAs**	miRBase database (Release 22)^19^;
**rRNAs, Y RNAs**	precompiled data from NCBI provided by SPORTS (version 1.1)^14^;
**tRNAs**	Genomic tRNA database (date: 25.05.2021)^23^;
**tRNAs**	tRF-1 and tRNA-leader sequence data (date: 09.04.2019)^12^;
**piRNAs**	piRNA cluster database (date: 25.05.2021)^24^;
**lncRNAs, sRNAs, snoRNAs**	Ensembl (Release 104)^25^;
**snoRNAs**	snoDB (version 2.0)^26^;

### Quality filtering of sequencing data

As quality control, the normalized data was converted to a two-dimensional T-distributed Stochastic Neighbor Embedding (t-SNE) space using the SCANPY toolkit^16^. When plotting the two embedding vectors against each other, four samples did not cluster with the samples of the same blood component type (0.8% of derivatives, 4/514) and were excluded from further analysis (Fig. 2). An overview on the age and gender distribution for the remaining 510 samples passing quality filtering is listed in Table 5.

**Table 5:** Sample overview showing the age and gender distribution for each blood component type after quality filtering.

	N	Gender, n (%)		Age, mean (SD)
		Female	Male	
**Overall**	510	300 (59%)	210 (41%)	62 (8)
**B cells**	51	29 (57%)	22 (43%)	63 (8)
**Basophils**	36	21 (58%)	15 (42%)	62 (8)
**CD4+ T cells**	50	30 (60%)	20 (40%)	63 (8)
**CD8+ T cells**	49	30 (61%)	19 (39%)	63 (8)
**Eosinophils**	46	28 (61%)	18 (39%)	63 (8)
**Erythrocytes**	49	29 (59%)	20 (41%)	63 (8)
**Monocytes**	47	28 (60%)	19 (40%)	62 (8)
**NK cells**	48	27 (56%)	21 (44%)	62 (8)
**Neutrophils**	51	29 (57%)	22 (43%)	63 (8)
**Plasma**	39	23 (59%)	16 (41%)	62 (8)
**Thrombocytes**	44	26 (59%)	18 (41%)	62 (8)

### Relative contribution of blood components to sRNA profile of whole blood

To estimate the contribution of the major peripheral blood cell types and plasma to the global sRNA profile of whole blood, we determined for each purified blood component fraction its sRNA content per microliter blood.

The sRNA content of the purified fraction of cell type *c* from donor *d* (*α*_*c,d*_) was calculated (Eq. (1)) as the product of the sRNA concentration (*c*_sRNA_; 10 to 200 nucleotide region of the Fragment Analyzer), the elution volume of the sorted cells (*V*_elution_) and the donor-specific blood count (n_blood_count_) divided by the number of sorted cells (*n*_sorted_count_):

1 $${\alpha }_{c,d}=\frac{{c}_{sRNA}\ast {V}_{elution}\ast {n}_{blood\_count}}{{n}_{sorted\_count}}$$

The sRNA content of the plasma fraction from donor *d* (*α*_*d*_) was calculated (Eq. (2)) as the product of the sRNA concentration (c_sRNA_) and the elution volume (V_elution_) of the isolated plasma RNA divided by the plasma volume used for RNA isolation (V_input_), which was then adjusted for the volume fraction of plasma in blood (0.5 was assumed):

2 $${\alpha }_{d}=\frac{{c}_{sRNA}\ast {V}_{elution}}{{V}_{input}}\ast 0.5$$

The calculated sRNA content values per sample can be found in Supplementary Table 3. For 15 of the 510 QC-filtered blood component specific samples (3%), the sRNA content could not be determined. For the remaining samples the sRNA expression (RPM values) of each sRNA *s* in each blood component *b* (*x*_*b,s,d*_) was scaled by multiplying the sample-specific sRNA content to account for the different cell counts and sRNA content per blood component. For each blood component *b*, the mean of these scaled RPM values per sRNA *s* is calculated (Eq. (3)) over all donors *D*:

3 $${\bar{x}}_{b,s}=\frac{1}{D}\left(\mathop{\sum }\limits_{d=1}^{D}{x}_{b,s,d}\ast {\alpha }_{b,d}\right)$$

Finally, the proportion of the scaled mean expression values (*P*_*b,s*_) of each blood component *b* was calculated (Eq. (4)) per sRNA *s*:

4 $${P}_{b,s}=\frac{{\bar{x}}_{b,s}}{{\sum }_{b=1}^{B=11}{\bar{x}}_{b,s}}$$

**Fig. 7:**
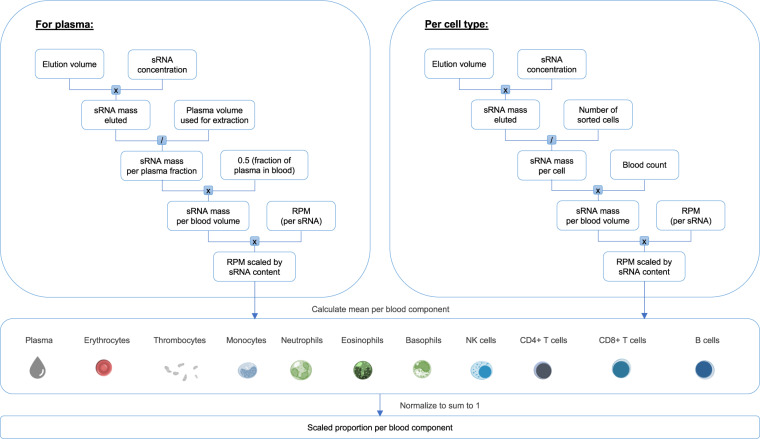
Outline of the calculations behind the deconvolution analysis to estimate the relative contribution of blood components to the sRNA profiles of whole blood. For each purified blood component specific sample, the eluted sRNA mass was calculated as the product of the sRNA concentration with the elution volume of the sample after purification. The eluted sRNA mass was then divided by the number of sorted cells to obtain the average sRNA mass per cell. For plasma samples, the volume inserted to extract the sRNAs was used to calculate the sRNA mass per plasma fraction. The sRNA mass per blood component sample was further multiplied by the blood count of the respective cell type to obtain for each cellular fraction the sRNA mass per blood volume. For plasma samples it was assumed that they make up half of the blood volume. The small RNA content was then used to scale the RPM values of the blood component specific samples obtained by sRNA sequencing. As a next step, the average scaled RPM values was calculated for each blood component type. Per sRNA these mean values per blood derivative were finally normalized to sum up to 1. These values reflect the proportional contribution of the distinct blood components to the global expression profile of a specific sRNA in a whole blood sample.

Figure 7 summarizes the deconvolution calculations graphically.

### Correlation analysis of whole blood sRNA expression and (sub-)cell type blood count

Whole blood sRNA expression can either be an indirect measure of the abundance of a certain blood component that is predominantly expressing this sRNA, indicate a regulation change of a cellular pathway that involves that sRNA or reflect both a combination of cell count changes and pathway regulation. To check whether the expression of a sRNA in whole blood correlates with the abundance of a certain cell type, we calculated Pearson correlation coefficients for RPM values of each sRNA and the blood counts of the major peripheral blood cell types and multiple leukocyte subpopulations.

### sRNA profile of blood components

To obtain relative sRNA profiles per blood component, for each sRNA the mean of the RPM values was calculated per blood component and divided by the sum of all mean RPM values per blood component. sRNAs that make up less than 2% of the sRNA profile were combined as ‘others’. To obtain blood component specific sRNA profiles aggregated per sRNA class, the RPM values of all sRNAs belonging to the same sRNA class were summed up per sample before calculating the mean per blood component.

### Identification of overrepresented sRNAs for blood components

To identify sRNAs that are overrepresented in a specific blood component, we used the differential expression testing method ‘rank_genes_groups’ from the SCANPY toolkit^16^. For each group of blood components, the expression distribution of a sRNA is compared against the expression distribution in all other blood components using the Wilcoxon rank-sum test. Only sRNAs with an increased fold change and adjusted p-value < 0.05 that are expressed in all samples of the respective blood component are considered as overrepresented.

### Comparison to previous benchmark

The raw sequencing data from the IKMB catalogue^10^ was downloaded from the National Center for Biotechnology Information (NCBI) Gene Expression Omnibus (GEO)^17^ and subjected to the same raw data processing and annotation as the *miR-Blood* dataset. For each sRNA name, the fraction of samples per blood component type with non-zero expression was checked. Only sRNAs that were detected in all samples of a blood component type were considered as detected in the respective dataset and used for the intersection analysis visualized by UpSet plots. To compare the expression correlation for matching blood component types, the mean log-transformed expression of each sRNA name was calculated per dataset. These means were then visualized as scatter plots and used to calculate the Pearson correlation coefficient *r* for each blood component type.

### Dashboard implementation

The dashboard was implemented as a Python-based Dash app using the Dash libraries Core Components, HTML Components, DAQ and Bio in addition to the Python graphing library Plotly with the module Plotly Express. The dashboard is accessible via this link http://mir-blood.com/.

## Data Records

Raw sequencing data as well as the processed expression matrices per sRNA name have been uploaded to the GEO database under the accession number GSE225872^18^.

## Technical Validation

### Quality control of cell purification

For each purified cellular fraction, the purity of isolated cells was determined by flow cytometry using a small aliquot of the sample. For 25 cellular fractions (4.6% of derivatives, 25/539) the purification process did not meet the prespecified quality standard (purity > 70% as determined by flow cytometry). These samples were excluded from the dataset. Representative gating strategies for the flow cytometry quality control are shown in Figs. 5, 6.

### Quality control of sRNA sequencing

The quality of generated sRNA libraries was checked on a Fragment Analyzer (Agilent, Santa Clara, USA). Moreover, the normalized sequencing data was converted to a two-dimensional t-SNE space to identify samples that do not cluster with the samples of the same blood component type. Four samples (0.8% of derivatives, 4/514) were therefore excluded from further analysis (Fig. 2).

### Comparison of the expression data to the previous benchmark

**Fig. 8:**
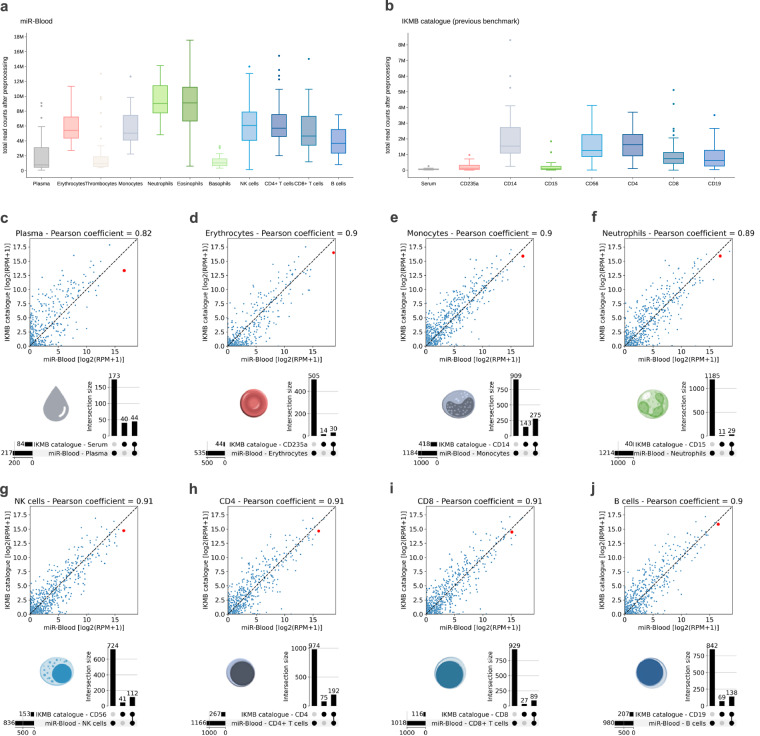
Comparison to previous benchmark. Total read counts after pre-processing for blood component-specific samples of (**a**) the *miR-Blood* dataset presented here and (**b**) the previous benchmark dataset^10^. (**c–j**) Direct comparison of samples per matching blood component type. The scatter plots show the mean expression of sRNAs that were detected in both datasets. Correlation coefficients are given. The outlier sRNA miR-16-5p is highlighted as a red dot. The UpSet plots below show the intersection of the subclass names of sRNAs with non-zero expression for all samples of a group (100% detection per group and dataset).

A comparison with the previous benchmark dataset, the IKMB catalogue^10^, showed a robust correlation (*r* ~ 0.9) of the mean expression of shared sRNAs per matched blood component group (Fig. 8c–j). This suggests a general comparability of the expression data. Discrepancies observed can be attributed to variations in sample processing (direct purification vs. pooled processing), library preparation methods (QIAseq vs. TruSeq), and sequencing depth (Fig. 8a,b). Due to the considerably higher sequencing depth in the *miR-Blood* dataset, normalized expression values tend to be lower compared to the IKMB catalogue.

As depicted in the UpSet plots (Fig. 8c–j), the *miR-Blood* dataset encompasses a significantly larger number of sRNA species compared to the previous benchmark. This is particularly pronounced in neutrophils, where 1214, as opposed to only 40, sRNAs are consistently detectable across all samples.

As noted previously^6^, the sequence of miR-16-5p can form an extremely stable stem loop with the sequencing library adapters used here, making it a preferred substrate for ligation and thus lead to an artificial overrepresentation (“jackpotting”) in the sequenced sRNA pool. Consequently, the abundance of reads mapping to miR-16-5p is increased throughout all blood component libraries. Compared to the benchmark dataset miR-16-5p remains to be the only detectable artefact (highlighted as red dot in Fig. 8c–j).

### Potential limitation

It should be noted that compared to the other blood cell types it was more challenging to purify sRNAs from basophiles, thrombocytes, and plasma. Consequently, much lower total read counts after pre-processing were obtained for the samples of these three blood components (Fig. 8a). The higher ratios of rRNA-derived sRNAs in these samples must therefore be considered at least partially caused by non-optimal RNA inputs during library preparation. As described above for miR-16-5p, library artefacts can distort the number of reads of a certain sRNA. We thus recommend orthogonal validation of expression via alternative methods such as quantitative PCR or northern blot analysis. In general, it should be noted that the addition of stabilization agents and different extraction and library preparation methods can lead to shifts in the detected expression. This should be considered when extrapolating from this dataset to differentially obtained whole blood expression profiles.

As a limitation of the deconvolution analysis, it must be noted that the contribution of plasma might be overestimated. In contrast to the blood cell types, where we experimentally determined the blood counts and sRNA content per cell, the relative blood fraction of plasma could only be estimated.

The processed expression matrices uploaded to GEO under accession number GSE225872^18^, which are also interactively browsable on the dashboard (http://mir-blood.com/), are based on miRBase^19^ for miRNAs, as this is still the reference used by most resources. Please note that the annotation as a true miRNA has been challenged for many of the sequences listed in miRBase in the last years^20^. Therefore, we strongly encourage users to check the miRBase identifiers for evaluation by the MirGeneDB team^21^.

### Supplementary information

Supplementary Table 1: Blood count data (number of cells per µl blood) for the 52 donors.

Supplementary Table 2: Cell sorting metrics of purified blood component samples per donor.

Supplementary Table 3: Calculated metrics used for deconvolution analysis.

## Data Availability

The code used for data pre-processing has been deposited on https://github.com/gitHBDX/mirblood-code. The following software versions were used: unitas v1.7.7, SeqMap v1.0.13, SPORTS v1.1, Bowtie v1.3, SCANPY v1.8.2, Python v3.10.6, Plotly v5.10.0, Plotly Express v0.4.1, SciPy v1.9.1, Seaborn v0.12.2, and UpSetPlot v0.8.0.
